# Therapeutic Effect of Mongolian Medicine RuXian-I on Hyperplasia of Mammary Gland Induced by Estrogen/Progesterone through CRYAB-Promoted Apoptosis

**DOI:** 10.1155/2020/5707106

**Published:** 2020-05-27

**Authors:** Jia Liu, Jun-Fei Zhang, Guo-Hua Gong, Bin Zhang, Cheng-Xi Wei

**Affiliations:** ^1^Medical Experimental Center, General Hospital of Ningxia Medical University, Yinchuan, Ningxia 750000, China; ^2^Department of Emergency Medical, General Hospital of Ningxia Medical University, Yinchuan, Ningxia 750000, China; ^3^Medicinal Chemistry and Pharmacology Institute, Inner Mongolia University for Nationalities, Tongliao, Inner Mongolia, China; ^4^Inner Mongolia Key Laboratory of Mongolian Medicine Pharmacology for Cardio-Cerebral Vascular System, Tongliao, Inner Mongolia, China; ^5^Affiliated Hospital of Inner Mongolia University for Nationalities, Institute of Mongolia and Western Medicinal Treatment, Tongliao, Inner Mongolia, China

## Abstract

The traditional Mongolian medicine (TMM) RuXian-I is an empirical formula specifically used for treating the hyperplasia of mammary gland (HMG) in clinic based on the principles of traditional Mongolian medicine, but the treatment mechanism is not completely clear. In this paper, we elaborated the mechanism of RuXian-I in the treatment of HMG induced by estrogen and progestogen from its toxicity and activity. Firstly, RuXian-I exhibited no toxic effect on HMG rats through no changes of body weight and food intake measurement and no pathologic changes of the organs (heart, liver, spleen, lung, and kidney) detected. Secondly, RuXian-I could decrease the increased nipple height and diameter and remarkably relieve the pathologic changes of HMG rats and also alleviate serum sex hormone levels (estradiol (E_2_), luteinizing hormone (LH), progesterone (P), and testosterone (T)) of HMG rats. Finally, RuXian-I could obviously inhibit the upregulation level of antiapoptotic protein CRYAB of HMG rats and promote mammary gland cell apoptosis of HMG rats via increases of promoting apoptosis protein caspases-3, 8, and 9 and Bax and tumor suppressor protein p53, decreases of antiapoptosis protein Bcl-2, and release of cytochrome c. These results suggested that RuXian-I has protective and therapeutic effects on HMG rats induced by estrogen and progestogen possibly via promoting apoptotic pathway regulated by CRYAB and is a promising agent for treating HMG.

## 1. Introduction

Hyperplasia of mammary gland (HMG) is the most common breast disease in middle-aged women worldwide [1]. Susceptibility of HMG is related to many factors, including menstrual cycle, breastfeeding, occupation, abuse of sex hormone agents, diet, and mental pressure [2]. In recent years, the number of patients with the noncancerous benign diseases is increasing, and the morbidity is enhancing quickly, with a much higher risk of causing mammary carcinoma [3]. Unfortunately, they are easily neglected because much more attentions have been paid to malignant lesions of the breast, breast cancer, for instance [4]. Therefore, attention to breast hyperplasia is imminent. And it is significant for human health to discover more convenient, cheaper, and effective new therapeutic remedies with few side effects for treating HMG and to explore the anti-HMG mechanisms of these remedies for blocking its development to breast cancer.

Growing attention to treat both breast cancer and HMG has been paid to chemical agents, including estrogen therapy [5], and surgical excision [6]. Surgical treatment of patients is generally difficult to be accepted, while chemical agents always bring many side effects and complications and high recurrence rate. However, a great deal of research has been conducted on the traditional Mongolian medicinal formula in Inner Mongolia of China, which has unique treatment methods in many diseases [7]. Mongolian doctors believe that the breast hyperplasia disease belongs to the Mongolian medical “Qi su bu ri le du sen” and “He yi bu ri le du sen” disease categories. The cause is mainly due to menstruation, especially postpartum, inadequate diet, improper living, or disorderly use of drugs and poor lactation, etc., which cause the imbalance of the three roots of He yi, Sheila fever, Yellow Water, and Ba da gan in the body, affecting the normal decomposition of essence and dregs. This produces diseased blood, Sheila fever, and yellow water. Under the action of He yi, the breast was swollen to form the breast “Qi su bu ri le du sen” disease. The main clinical manifestations are a lump, pain, nipple discharge, and irregular menstruation, upset and chest tightness, insomnia, and more dreams. The disease is prone to recurrence and persistence, which seriously affects women's physical and mental health. Therefore, “Pingqi blood circulation and Huoxue Tongluo” have curative effects on breast hemorrhage and mastoma of gynecology. The traditional Mongolian medicine RuXian-I is also known as “Hu hun e ru le” which consists of 30 classic Mongolian medicines. It has the functions of calming Qi and blood, promoting blood circulation, resolving essences, and dross decomposition. It can promote the blood flow of the body and has the effects of regulating menstruation, activating blood, analgesic, swelling, soothing the nerves and nourish. Therefore, it has a very good effect on breast hyperplasia in women [8, 9]. Although there is a good effect, little is known about the mechanism.

The previous study has revealed RuXian-I in the treatment of HMG affected the expression of seventeen proteins identified via using proteomics. Among these, RuXian-I significantly downregulated the expression level of crystallin alpha B (CRYAB) in HMG rats [9]. CRYAB is a member of the small heat-shock protein family (sHsps) and highly expressed in the lens and to a lesser extent in several other tissues, including heart, skeletal muscle, and brain [10]. Recently, CRYAB has been found to exert multiple functions. In which, one of the most important function processes is the resistance to apoptosis induced by various stress factors, including oxidative stress [11] and endoplasmic reticulum stress [12]. When CRYAB is resistant to apoptosis in the selection of cells, HMG forms and, when it promotes oncogenic transformation, even leads to the onset of mammary tumors [13]. Therefore, the treatment mechanism of RuXian-I may be mediated by regulating the association between the CRYAB protein and HMG and is associated with apoptosis. However, the specific mechanism is not clear in anti-HMG effect of RuXian-I. So in order to further elucidate the mechanism, this study replicated the experiment of RuXian-I in the treatment of HMG again and focused on the relevant content of the expression of CRYAB and apoptosis *in vivo*.

## 2. Materials and Methods

### 2.1. Chemicals and Reagents

RuXian-I was provided by the Mongolian Medicine Manufacturing Room of the Affiliated Hospital of Mongolia University for Nationalities. All medicinal materials were purchased by Anhui Zhongmeitang Chinese Herbal Pieces Co., Ltd (NM2016-12). Estradiol benzoate injection and progesterone injection were bought from Hangzhou Animal Medicine Factory and Zhejiang Xianju Pharmaceutical Co., Ltd, respectively. Olive oil was bought from Sigma-Aldrich. Commercial Mitochondria/Cytosol Fractionation Kit following manufacturer's protocol was purchased from BioVision Inc., Mountain View, CA. Bicinchoninic acid (BCA) protein assay kit was purchased from Beijing Tiangen Biotech Co., Ltd. Rabbit anti-CRYAB, rabbit anti-caspase-3, mouse anti-caspase-9, rabbit anti-caspase-8, and rabbit anti-COX IV were purchased from Cell Signal Technology, Inc. Rabbit anti-Bax and rabbit anti-Bcl-2 were purchased from Abcam. Rabbit anti-cytochrome c was purchased from Santa Cruz Biotechnology, Inc. Rabbit anti-p53 and mouse anti-GAPDH were purchased from OriGene Technologies, Inc. Peroxidase-conjugated Affinipure goat anti-rabbit IgG and rabbit anti-mouse IgG were purchased from Beijing fir Zhongshan Goldenbridge Biotechnology Corporation. Immobilon^TM^ Western Chemiluminescent HRP Substrate and polyvinylidene fluoride (PVDF) membrane (0.22 *µ*m) were purchased from Millipore. Progesterone (P), estradiol (E_2_), prolactin (PRL), follicle-stimulating hormone (FSH), luteinizing hormone (LH), and testosterone (T) ELISA kits were bought from the Nanjing Jiancheng Bioengineering Institute. All other reagents were of analytical grade and were purchased from Amersham Biosciences (Uppsala, Sweden).

### 2.2. Animal Models and Treatments

Fifty virgin female Wistar rats weighing 200 ± 20 g were supplied by the experimental animal center of Jilin University (SCXK-2016-05). The rats were raised in plastic cages with temperature- and humidity-controlled room (20°C–25°C and 50%–60%, respectively) under a 12 h light/dark cycle (the illumination time: 6:00∼18:00), with rodent chow and water *ad libitum*, acclimation for a week. All procedures in the present study were performed in accordance with the Guide for the Care and Use of Laboratory Animals as adopted by the National Institutes of Health (Washington DC, USA, 1996). Local ethical committee approval was also obtained (Ethics Committee number: 0010/2016). All efforts were made to minimize animal suffering and to reduce the number of animals used. The rats were randomly assigned into 5 groups (*n* = 10). Ten rats as the normal control group were injected with normal saline (0.25 mL·kg^−1^, i.m.) for 30 days, and the remaining were injected with estradiol benzoate (0.5 mg·kg^−1^, i.m.) for 25 days, and followed with progesterone (5 mg·kg^−1^, i.m.) for 5 days. Then, rats in the normal control group and HMG control group were received normal saline (10 mL·kg^−1^, i.g.) for 30 days, while rats in the low-dosage, middle-dosage, and high-dosage treatment groups were treated with RuXian-I (0.5 g·kg^−1^, 1.5 g·kg^−1^, and 3.0 g·kg^−1^, respectively, i.g.) [8, 9] for 30 days.

### 2.3. Body Weight, Food Intake, and the Nipple Height and Diameter

During the experiment, the rats' weight and the second pair of nipple diameters and height were measured and recorded on the day before the experiment, the last day of making the model, and the seventh day of treatment per week until termination. Food intake was measured and recorded on every day of administration, and the average intake continuously weekly is taken as the change in food intake until termination.

### 2.4. ELISA

After termination, the rats were euthanized for 1 h after the last administration of drugs, and the blood samples were collected from abdominal aortic. The serum samples were obtained by centrifugation at 3500 rpm for 10 min after coagulated at 4°C overnight. The serum samples were used for the determination of the levels of sex hormone and stored at −80°C until biochemical assays were performed. Rat serum P, E_2_, PRL, FSH, LH, and T concentrations were measured. Enzyme-linked immunosorbent assay (ELISA) kits were used for all measurements according to the procedures recommended by the manufacturer.

### 2.5. Histopathology Observation

Tissues between the second and the third nipples on the right side from all groups were separated and fixed in 4% paraformaldehyde, embedded in paraffin, sectioned into 4 *µ*m pieces, stained with haematoxylin-eosin (H&E), and observed under light microscopy, and photos were taken and analyzed.

### 2.6. Immunohistochemistry Observation

Histological sections were immunostained using anti-CRYAB (1 : 1000) and/or PRDX1 (1 : 1000) antibodies. Peroxidase-conjugated Affinipure goat anti-rabbit IgG (1 : 500) was used as secondary antibodies. Haematoxylin was used for counterstaining. Then, sections were observed under light microscopy, and photos were taken and analyzed.

### 2.7. Western Blot Assay

The mammary gland tissues were rapidly separated on ice plate, frozen in liquid nitrogen, and stored at −80°C. Mitochondrial and cytosolic proteins were extracted from tissues of the mammary gland randomly selected from each group and isolated using a commercial Mitochondria/Cytosol Fractionation Kit following the manufacturer's protocol. Their protein content was detected by BCA protein assay kit. Total protein (20 *µ*g) per sample was resolved by 12% sodium dodecyl sulfate-polyacrylamide gel electrophoresis (SDS-PAGE) and transferred to PVDF membrane. CRYAB (1 : 1000), caspase-3 (1 : 1000), caspase-8 (1 : 1000), caspase-9 (1 : 1000), Bax (1 : 1000), Bcl-2 (1 : 200), p53 (1 : 1000), cytochrome c (1 : 100), and COX IV(1 : 1000) antibodies were added and then the corresponding peroxidase-conjugated Affinipure goat anti-rabbit IgG (1 : 5000) and rabbit anti-mouse IgG (1 : 5000) secondary antiserum were detected. Chemiluminescent HRP substrate was detected and PVDF membrane was developed.

### 2.8. Statistical Analysis

All values were expressed as the means ± SD. Data were analyzed using the SPSS17.0 statistical software. Data among the groups were analyzed by the homogeneity test of variances expressed in forms of standard deviation. Statistical analysis for multiple comparisons was performed using one-way ANOVA followed by post hoc test. All experiments were performed at least three times. *P* < 0.05 was accepted as significant. The statistic and graphic software GraphPad Prism (version 5, GraphPad Software, Inc., La Jolla, CA) was used for all statistical and graphic analysis.

## 3. Results

### 3.1. RuXian-I Had No Toxic Effect on HMG Rats

**Figure 1:**
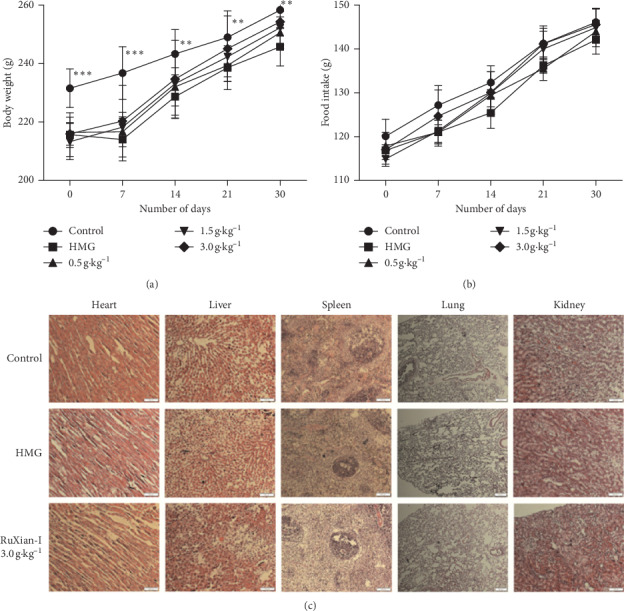
Changes of average body weight (a) and average food intake (b) with time in each group and histopathological changes of heart, liver, spleen, lung, and kidney in the control group, HMG group, and RuXian-I high-dose group. ((c) original magnification, 400×). ^*∗∗*^*P* < 0.01 and ^*∗∗∗*^*P* < 0.001 vs. the HMG group.

The rat body weight and food intake during the administration were measured, and histopathology was performed for toxicity assessment in the control group, HMG group, and all groups treated by RuXian-I. The body weight in the HMG group during treatment decreased remarkably by estrogen and progestogen induction (Figure 1(a)). The average body weight in RuXian-I groups showed a small but no significant dependent changes with increased dose of RuXian-I treatment, and at the third and fourth weeks of treatment, that in the high-dosage group (3.0 g·kg^−1^) was close to the control group. There was no significant difference in the average food intake of control model, HMG group, and all groups treated by RuXian-I during treatment (Figure 1(b)).

H&E staining showed there was no significant difference in the five main organs, heart, liver, spleen, lung, and kidney, of control rats, HMG rats, and RuXian-I-treated (3.0 g·kg^−1^) rats (Figure 1(c)). The above results indicated that RuXian-I has no toxic effect on HMG rats.

### 3.2. RuXian-I Reduced the Nipple Height and Diameter of HMG Rats

**Figure 2:**
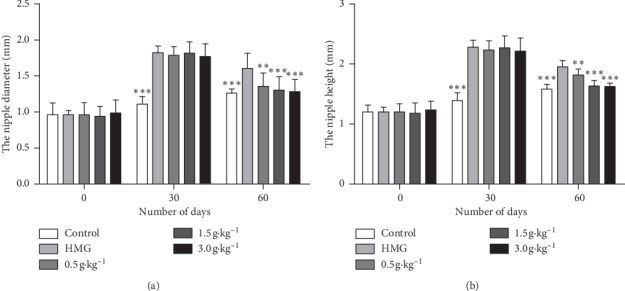
The nipple diameter and height changed on the 0th, the 30th, and the 60th day of the administration in each group. ^*∗∗*^*P* < 0.01 and ^*∗∗∗*^*P* < 0.001 vs. the HMG group.

The nipple height and diameter were measured on the 0th day, the 30th day, and the 60th day of the administration. The nipple height and diameter of rats were enlarged in the HMG model group and all RuXian-I groups after induction by estrogen for 25 days and progestogen for 5 days compared with the control group (*P* < 0.001, Figure 2), and there was no difference among the four groups (Figure 2), while these two indicators showed a dose-dependent alleviation after treatment by RuXian-I for 30 days compared with the HMG group (*P* < 0.01 or *P* < 0.001, Figure 2).

### 3.3. RuXian-I Alleviated Serum Sex Hormone Levels of HMG Rats

**Figure 3:**
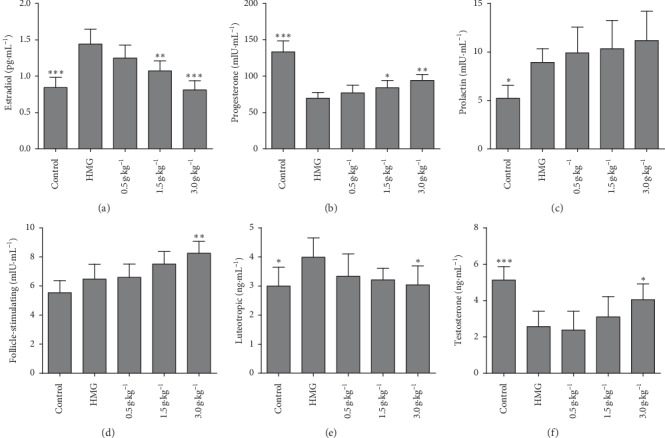
Serum sex hormone levels of each group from three independent experiments. ^*∗*^*P* < 0.05, ^*∗∗*^*P* < 0.01, and ^*∗∗∗*^*P* < 0.01 vs. the HMG group. (a) E_2_, (b) P, (c) PRL, (d) FSH, (e) LH, and (f) T.

To assess changes of serum sex hormone, 6 sex hormone levels were tested by ELISA. Compared with the control group, in the rat plasma of HMG disease model, E_2_ and LH concentrations were remarkably increased (Figure 3); PRL and FSH concentrations were insignificantly elevated (Figure 3); P and T concentrations were remarkably decreased (Figure 3). Compared with the HMG group, in all groups treated by RuXian-I, E_2_ and LH levels showed a decreasing dose dependency of RuXian-I treatment (Figure 3), while P and T levels showed an increasing dose dependency of RuXian-I treatment (Figure 3). Such a response of PRL and FSH levels of HMG rats had not been reduced by treatment with RuXian-I, but increased (Figure 3).

### 3.4. RuXian-I Improved Mammary Gland Histopathologic Changes of HMG Rats

**Figure 4:**
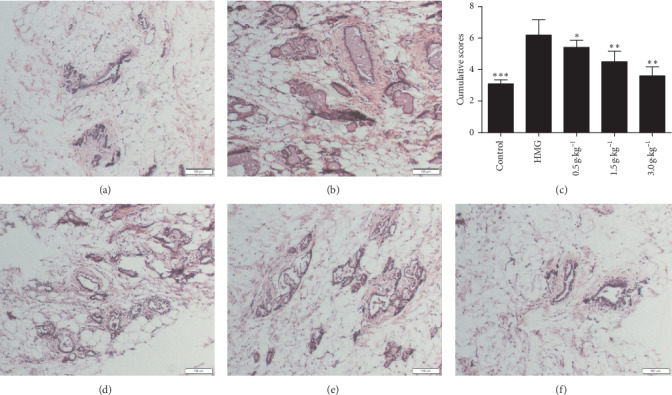
Histological image of mammary gland tissue in each group (original magnification, 400×). Bar chart indicates pathological graded score. ^*∗*^*P* < 0.05, ^*∗∗*^*P* < 0.01, and ^*∗∗∗*^*P* < 0.001 vs. the HMG group. (a) Control. (b) HMG. (c) Grade scores. (d) 0.5 g·kg^−1^. (e) 1.5 g·kg^−1^. (f) 3.0 g·kg^−1^.

To evaluate the degree of hyperplasia for HMG rats, H&E staining was performed on mammary gland sections of each group. The rat mammary gland in the control group showed normal histological architecture with ducts and acini. The very small acini were arranged closely around and no expansion into the scattered small- and medium-sized mammary ducts, showing a single layer and sometimes bilayer of cubical epithelium. Ducts and acini were surrounded by an abundant content of adipose connective tissues in which were capillary networks (Figure 4).

The rat mammary gland in the HMG group showed an increase in the number and size of ducts and acini. Most shedding epithelium and secretions in the lumen and the ductal walls were significantly thickened and the cells had layers. This was accompanied by a large amount of epithelial cells irregularly arranged, and lobular expansion in the acinar chamber and stromal hyperplasia (Figure 4).

In all RuXian-I-treated groups, the number and size of rat mammary gland lobules and acini and cavity secretions were less than the HMG group. Catheter lumen and volume became smaller in the treatment group. Especially in the high-dosage group (3.0 g·kg^−1^), the number of lobules and acini was significantly reduced and there was most cubic epithelium in acini, with a few flat, no secretions within the cavity or a little. These results indicated that RuXian-I showed dose dependency in improving mammary gland histopathologic changes of HMG rats (Figure 4).

### 3.5. RuXian-I Downregulated CRYAB Level of HMG Rats

**Figure 5:**
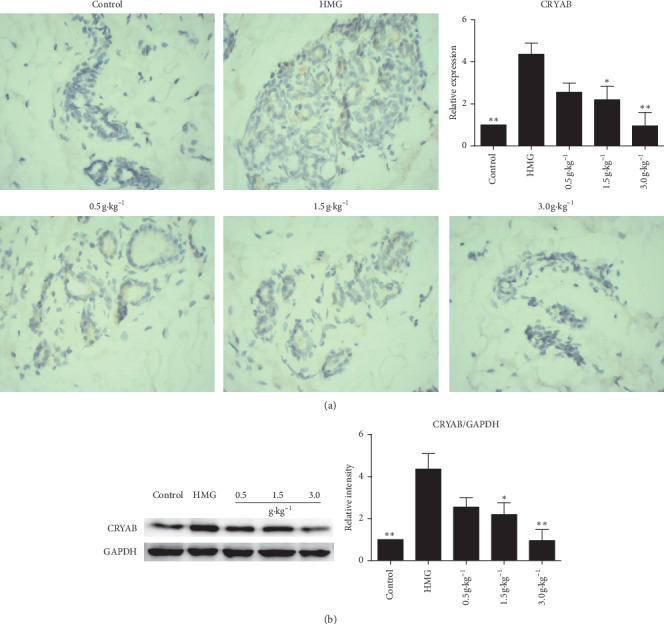
Expression of CRYAB protein in breast tissues of each group. Bar graph showing the relative expression of brown particles. (a) Immunohistochemistry results (original magnification, 400×). (b) Western blotting results. The left panel shows a western blot from a representative experiment. The right panel shows the fold changes calculated by normalization of band density with GAPDH from three independent experiments. ^*∗*^*P* < 0.05 and ^*∗∗*^*P* < 0.01 vs. the HMG group.

To determine the expression of *α*B crystallin protein in the anti-HMG effects of RuXian-I, immunohistochemistry and western blotting were performed to check its level. Immunostained mammary gland sections with CRYAB showed a few epithelial cells exhibiting an obvious positive cytoplasmic reaction in the form of a brown color in the HMG group in comparison with the control group. In all RuXian-I-treated groups, many epithelial cells exhibited a positive cytoplasmic CRYAB immunostaining (Figure 5(a)).

Western blotting results also showed that the expression of CRYAB in the HMG group was distinctly upregulated (Figure 5(b)), while this effect was attenuated by dose dependency of RuXian-I, especially significantly downregulated in high-dosage (3.0 g·kg^−1^) group (Figure 5(b)).

### 3.6. Effect of RuXian-I on Expression of Mitochondrion-Related Protein in Breast Tissue of HMG Rats

**Figure 6:**
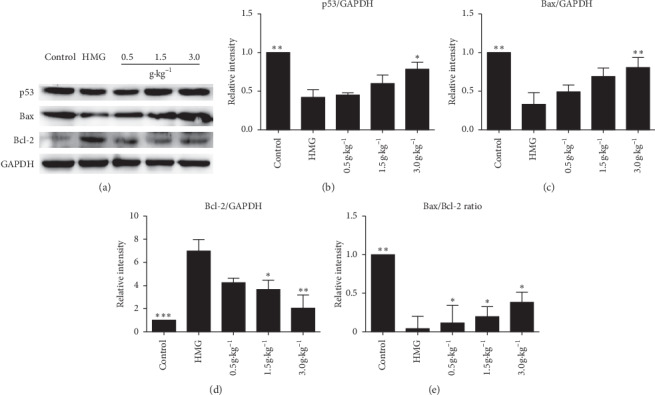
Mitochondrial-associated proteins p53, Bax, and Bcl-2 were analyzed on the same immunoblot from each group. Histogram shows the fold changes calculated by normalization of band density with GAPDH from three independent experiments. Normalization for Bax and Bcl-2 against GAPDH was made before calculating the Bax/Bcl-2 ratio. ^*∗*^*P* < 0.05 and ^*∗∗*^*P* < 0.01.

To investigate the possible molecular mechanism of RuXian-I treatment in HMG rats, western blot was used to detect the expression of mitochondrial-associated proteins p53, Bax, Bcl-2, and cytochrome c in mammary glands. The results showed that, compared with the control group, the expression of p53 and Bax was significantly downregulated (Figure 6), while Bcl-2 was significantly upregulated (Figure 6). However, compared with HMG, the expression of p53 and Bax showed a dose-dependent upregulation, while Bcl-2 showed a dose-dependent downregulation. The ratio of Bax/Bcl-2 expression was analyzed to evaluate the effect of RuXian-I on the activation of caspase. Compared with the control group, the ratio of Bax/Bcl-2 in the HMG group was reduced by about 21 times, while the ratio after the RuXian-I treatment was significantly increased (Figure 6). It shows that RuXian-I can achieve the purpose of treating HMG by activating apoptosis.

**Figure 7:**
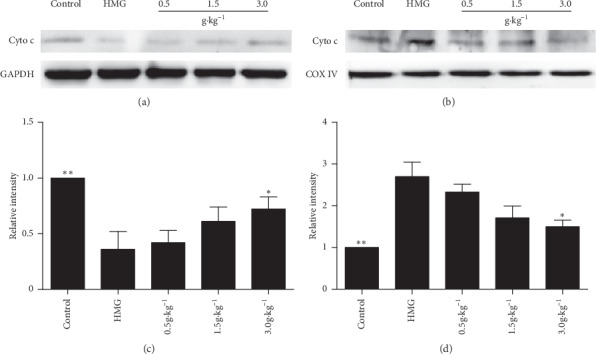
Cytochrome c releasing from mitochondria was analyzed on the same immunoblot from each group. Histogram shows the fold changes calculated by normalization of band density with GAPDH from three independent experiments. ^*∗*^*P* < 0.05 and ^*∗∗*^*P* < 0.01. (a) Cytosolic fraction. (b) Mitochondrial fraction. (c) Cyto c/GAPDH. (d) Cyto c/COX IV.

The cytochrome c results showed that, compared with the control group, the expression of cytochrome c in the HMG group was significantly increased in the mitochondria (Figure 7) and significantly decreased in the cytoplasm (Figure 7), but after treatment with RuXian-I, the expression of cytochrome c was reduced in the mitochondria and increased in the cytoplasm, both in a dose-dependent manner (Figure 7). It demonstrated that cytochrome c accumulated in mitochondria after HMG, while cytochrome c released from mitochondria to cytoplasm after treatment with RuXian-I.

### 3.7. Effect of RuXian-I on Apoptosis-Associated Protein Levels in Mammary Glands of HMG Rats

**Figure 8:**
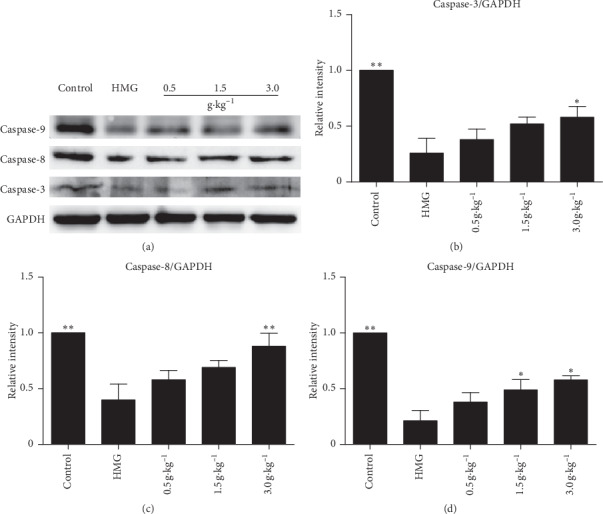
Caspases-3, 8, and 9 were analyzed on the same immunoblot from each group. Histogram shows the fold changes calculated by normalization of band density with GAPDH from three independent experiments. ^*∗*^*P* < 0.05 and ^*∗∗*^*P* < 0.01.

Then, the expression levels of caspases-3, 8, and 9 in mammary tissues were detected. The results showed that, compared with the control group, the expression of caspases-3, 8, and 9 in rats induced by estrogen and progesterone was significantly lower in the HMG group (Figure 8). However, there was a dose-dependent increase in protein expression in hormone-induced rats treated with RuXian-I, especially in the high-dose group of RuXian-I (Figure 8). This shows that treatment of HMG by RuXian-I is closely related to apoptosis.

## 4. Discussion

In the present study, no toxic effect of RuXian-I was found on HMG rats at low, middle, and high dose for 30 days by no changes of body weight and food intake measurement and no pathologic changes of the five main organs detected, and RuXian-I could relieve the heights and diameters of nipples and eliminate the hyperplasia of lobules and gland alveolus in different degree. Furthermore, HMG is a kind of pathological hyperplasia of mammary gland lobules induced by the balance disorder of estrogen and progesterone [14], controlled and secreted abnormally by hypothalamus-pituitary-gonadal axis. When extraneous factors act on the human body, sex hormone secretion becomes abnormal leading to the occurrence of a variety of diseases such as hyperprolactinemia, HMG, hysteromyoma, and infertility. In mammals, PRL can regulate mammary glandogenesis, promote milk secretion, and trigger and maintain lactation through autologous and paracrine as a cytokine [15, 16]. Our previous research revealed that PRL played a very important role in the formation of HMG [9]. However, the exact mechanism of PRL in the action of HMG is not clear. Our study indicates RuXian-I could regulate the sex hormone disorder, including P, E_2_, PRL, FSH, LH, and T levels, possibly by adjusting the balance of hypothalamus-pituitary-gonadal axis. These results showed that RuXian-I has therapeutic effect and no toxic effect on HMG rats induced by estrogen and progestogen.

Our study also showed the high expression of CRYAB, the major small heat-shock protein, is associated with HMG. CRYAB plays a crucial role in the development of diverse diseases. It, as a chief regulator, regulates cardiomyocyte apoptosis during hypertrophy and myocardial infarction [17] and targets to prevent posterior capsule opacification [18]. Loss of CRYAB in the human lens epithelium could be associated with age-related and congenital cataracts [19]. However, high expression of CRYAB also involves in multiple diseases. For instance, it is highly expressed in the substantia nigra of Parkinson's disease (PD) brain and may be involved in the glial pathology during dopaminergic neuron degeneration in PD [20]. In recent years, the role of CRYAB as a marker in tumorigenesis, tumor invasion, and metastasis and prognosis has received increasing attention. CRYAB is highly expressed in a variety of cancers, among which includes breast cancer [21], lung cancer [22], colorectal cancer [23], thyroid carcinoma [24], and glioblastoma [25]. In the present study, CRYAB is significantly highly expressed in HMG model rats.

**Figure 9:**
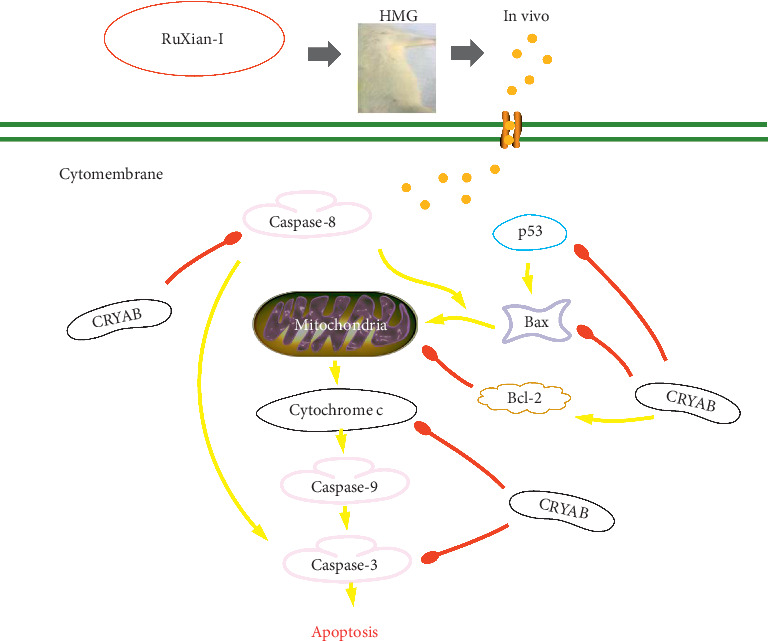
A schematic of the mechanisms by which CRYAB protein controls apoptosis and survival in RuXian-I-treated HMG. Red arrow: inhibition; yellow arrow: stimulation.

CRYAB functions as a molecular chaperone involving in several cellular processes, such as signal transduction, protein degradation, stabilization of cytoskeletal structures, and apoptosis [26]. CRYAB as a molecular chaperone is also a well-known antiapoptotic polypeptide [27] whose major property is to negatively regulate apoptosis by directly binding to partially processed caspase-3 (p24 intermediate) and Bax and Bcl-X_L_ (two proapoptotic members of the Bcl-2 family), thus inhibiting the proapoptotic function of these proteins [28]. CRYAB also sequesters their translocation from the cytosol into mitochondria and thereby prevents stress-induced apoptosis 49. Similarly, CRYAB binds to p53, whose deletion suppresses the apoptotic pathway by suppressing Bax expression level [29], to sequester its translocation to mitochondria, and therefore indirectly inhibits its proapoptotic action towards antiapoptotic Bcl-2 molecules [30]. In addition to interacting with the above clients, CRYAB also negatively regulates caspase-8-dependent activation of caspase-3 by inhibiting its autoproteolytic maturation [31]. Needless to say, a low expression of CRYAB enhances mitochondrial dysfunction including mitochondrial depolarization, the enhancement of mitochondrial permeability, and release of cytochrome c [13, 14]. However, when CRYAB inhibits caspase activation to be resistant to apoptosis and promotes oncogenic transformation in the selection of cells, HMG forms and even leads to the onset of mammary tumors [15]. In our study, antiapoptotic protein CRYAB and protein Bcl-2 were increased, while promoting apoptosis protein caspases-3, caspases-8, caspases-9, and Bax, tumor suppressor protein p53, and cytochrome c were decreased. However, RuXian-I could inhibit the upregulation level of recognized antiapoptotic protein CRYAB, decrease antiapoptotic protein Bcl-2, increase the promoting apoptosis protein caspases-3, caspases-8, caspases-9, and Bax and tumor suppressor protein p53 and cause the release of cytochrome c, suggesting that RuXian-I has protective and therapeutic effects on HMG rats induced by estrogen and progestogen possibly via promoting breast cell apoptosis pathway regulated by CRYAB and is a promising agent for treating HMG. The possible mechanisms are shown in Figure 9. The exact mechanism of apoptosis regulated by CRYAB protein needs further study in the mammary gland tissue of HMG treated by RuXian-I.

## 5. Conclusion

We report for the first time the role of antiapoptotic protein CRYAB in regulating apoptosis in HMG. The mechanism of RuXian-I in treating HMG in rat may be the apoptosis induced by inhibiting CRYAB. CRYAB may be used as a target protein of HMG treatment. This paper lays a foundation for studying the mechanism of HMG and provides a scientific basis for the better application of RuXian-I in clinical practice.

## Data Availability

All data generated or analyzed during this study are included in this article. However, further details are available from the corresponding author on reasonable request.
